# Multicommutated Flow Analysis System for Determination of Horseradish Peroxidase and Its Inhibitors †

**DOI:** 10.3390/molecules26185630

**Published:** 2021-09-16

**Authors:** Justyna Głowacka, Kamil Strzelak, Robert Koncki

**Affiliations:** Faculty of Chemistry, University of Warsaw, Pasteura 1, 02-093 Warsaw, Poland; kamil.strzelak@chem.uw.edu.pl (K.S.); rkoncki@chem.uw.edu.pl (R.K.)

**Keywords:** horseradish peroxidase, flow analysis, multicommutation, thiols

## Abstract

A fully mechanized multicommutated flow analysis (MCFA) system dedicated to determining horseradish peroxidase (HRP) activity was developed. Detection was conducted using a flow-through optoelectronic detector-constructed of paired LEDs operating according to the paired emitter-detector diode (PEDD) principle. The PEDD-MCFA system is dedicated to monitoring the enzyme-catalyzed oxidation of p-phenylenediamine (pPD) by a hydrogen peroxide. Under optimized conditions, the presented bioanalytical system was characterized by a linear response range (33.47–200 U/L) with a detection limit at 10.54 U/L HRP activity and 1.66 mV·L/U sensitivity, relatively high throughput (12 signals recordings per hour), and acceptable precision (RSD below 6%). Additionally, the utility of the developed PEDD-MCFA system for the determination of HRP inhibitors allowing the detection of selected thiols at micromolar levels, is demonstrated. The practical utility of the flow system was illustrated by the analysis of some dietary supplements containing L-cysteine, N-acetylcysteine, and L-glutathione.

## 1. Introduction

Modern analytical chemistry flow methods are still unequaled in terms of versatility and simplicity. Principles, advances, and applications of flow analysis systems have been reviewed recently [1,2,3,4]. Modern flow analysis techniques offer the mechanization and automation of the whole analytical procedures as well as an independent optimization of each step of multistep analytical protocols. Microprocessor-based controls of each stepwise operation provide a high measurement repeatability. The consumption of reagents and samples is being minimized, especially when flow analysis systems are downscaled to a meso/microfluidic format. Multicommutated flow analysis (MCFA) systems, designed with modular devices (microsolenoid pumps and valves), are relatively inexpensive, especially when compared to the costs of heavy analytical instrumentation. Flow analysis systems are predominantly intended to detect a single analyte; therefore, the development of dedicated flow-through detectors becomes obvious. For example, spectrophotometers and spectrofluorometers are being replaced by small-sized and highly economic optoelectronic detectors. In the following work, an extremely low-cost LED-based detector operating according to the paired emitter-detector diode (PEDD) principle [5,6] has been applied.

Flow techniques are especially attractive for kinetic-catalytic methods of analysis [7,8], where highly reproducible conditions of reagents dosing, mixing, and transport as well as the precise control of reaction/incubation times are crucial. Such conditions are especially important in the case of enzyme activity assays. Therefore, several flow analysis systems designed to detect various hydrolases, such as alkaline phosphatase [9,10], acid phosphatase [10], α-amylase [11], urease [12] cutinase [13], and β-galactosidase [14], have been developed. Flow analysis systems for the assaying of the activity of oxidoreductases (such as catalase [15], peroxidase [16], and ceruloplasmin [17]) are less often reported in the literature. In this work, a PEDD-MCFA system for the determination of horseradish peroxidase (HRP) activity is presented. Surprisingly, there is only one report of electrochemical HRP assay in the flow analysis format until now, which was published when this analytical technique was still in its infancy [18].

HRP (EC 1.11.1.7) plays a fundamental role in many fields, such as biotechnology [19], medicine [20,21], environmental protection [22], and the food industry [23], due to its numerous advantages including stability, availability, low cost, high sensitivity in analyte range detection as well as the ability to catalyze the oxidation reaction of a large group of chromogenic substrates [24,25]. Moreover, many compounds inhibiting its activity, such as thiols, have been reported in the literature and can be easily determined using a slightly modified enzyme assay. Thiols containing a sulfhydryl group (-SH) in their structure can be found in all living organisms due to their numerous functions. First of all, they are responsible for protecting cells against oxidative stress and its consequences, such as tissue damage or the initiation of many new diseases, caused by disturbances of the balance state between free radicals and antioxidants [26,27]. In addition, thiols enable to maintenance of both proper redox potential in cells, which is especially important in the regulation of intracellular metabolism and appropriate structure, as well as protein function [28]. Hence, their deficiency can cause brittle nails or hair and even the appearance of more serious diseases, such as cardiovascular diseases [29] and cancers [30].

The main objective of the presented research was to develop a simple and inexpensive strategy for the HRP activity and for the detection of its inhibitors. This paper presents a highly economic bioanalytical device for HRP assaying based on PEDD-detection coupled with the system of solenoid micropumps and microvalves actuated by an Arduino microcontroller. To examine the activity of HRP, a photometric method based on the oxidation of p-phenylenediamine (pPD) by hydrogen peroxide in the presence of enzymes [31] is used. The PEDD-MCFA system, developed for the HRP assays, has been easily adapted for the indirect inhibitive detection of selected mercaptocompounds. As the final step, the presented system was tested for use in dietary supplements analysis. 

## 2. Experimental

Horseradish peroxidase (powder, ≈150 U/mg, Cat. No. 77332) and p-phenylenediamine used as a substrate for enzymatic reaction (pPD, Cat. No. 78429) were purchased from Sigma-Aldrich (St. Louis, MO, USA). The hydrogen peroxide (Cat. No. 885196722) as well as other reagents of analytical grade were acquired from POCh (Gliwice, Poland). The enzymatic reaction was conducted in 100 mM phosphate buffer (pH = 6.5). All of the potential HRP inhibitors, reduced L-Glutathione (Cat. No. G4251), 2-mercaptoethylamine (Cat. No. M-6500), oxidized L-Glutathione (Cat. No. 49740), N-acetyl-l-cysteine (Cat. No. A-7250), D-cysteine hydrochloride monohydrate (Cat. No. C8005), L-cysteine hydrochloride monohydrate (Cat. No. C-7880), and sodium 2-mercaptoethanesulfonic (Mesna, Cat. No. M-1511), were purchased from Sigma-Aldrich (St. Louis, MO, USA). Doubly distilled water was used throughout all of the experiments.

All of the dietary supplements used in this work (Table S1 in the Supplementary Material) were taken from a local pharmacy. The content of each supplement dose was dissolved in 150 mL of distilled water (in the case of water-soluble samples) or 140 mL of distilled water and 10 mL of 0.1 mol/L HCl (in the case of samples insoluble in water) and were then intensively stirred for about an hour. To obtain clear solutions, some of them were filtered. The analyzed solutions were diluted 10-, 25- or 100-fold before the experiment. Thus, the effects related to color as well as the turbidity of some the samples were significantly reduced. Reference determinations of the thiols were performed by iodometric titration, according to the pharmacopeial protocol using 0.05 mol/L iodine in 0.24 mol/L of a potassium iodide solution and 0.1 mol/L of a sodium thiosulfate solution as titrants. All of the reagents used for iodometric titration were purchased from Sigma-Aldrich (St. Louis, MO, USA).

The Multicommutation Flow Analysis (MCFA) system was constructed from three-way solenoid microvalves (product no. 100T3MP12-62) and solenoid micropumps (indicated stroke volume of 20 µL, product no. 120SP1210-4TE) purchased from Bio-Chem Fluids (Boonton, NJ, USA). All of the tubing connecting the individual parts of the manifold were made of PTFE Microbore tubing (ID 0.8 mm), which was obtained from Cole-Palmer (Vernon Hills, IL, USA). All solenoid devices were controlled by the Arduino Mega 2560 microcontroller with the integrated circuit ULN 2803 (TME, Łódź, Poland).

The photometric detector was based on the paired emitter-detector diode principle and consisted of a 520 nm diode (Cat. No. OSPG53E1 A–MN, TME) as an emitter and a 630 nm diode (Cat. No. OSR5MA57E1 A–MN, TME) as a detector. The emitter diode was supplied by a stable current, which was provided by above-mentioned Arduino microcontroller. LEDs were placed in a flow-cell with an optical path length of 10 mm, a diameter of 3 mm, and a dead volume of approximately 70 µL. The measuring cell (Figure S1 in Supplementary Material) was made of a hard and chemically inert material—PEEK (poly-ether ether ketone, Plastic Group, Warsaw, Poland), by using a lathe and a milling machine. The electromotive force was an analytical signal generated by a detecting diode and was measured and then recorded using a multimeter (model UT70B, UNI-T) connected by a RS232 interface to a data storage computer.

## 3. Results and Discussion

**Figure 1:**
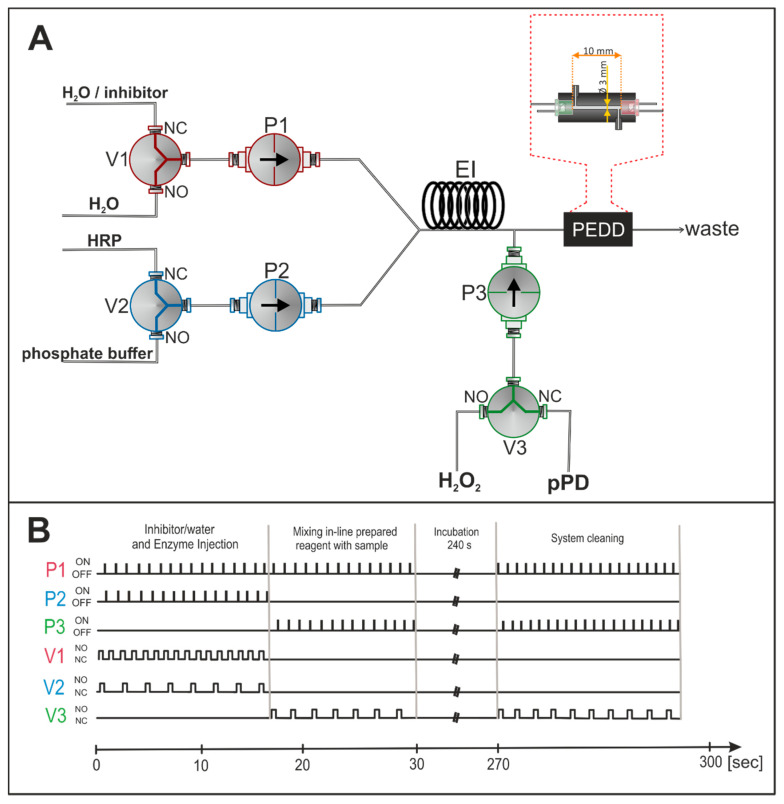
Scheme of multicommutation flow analysis system for the determination of both HRP enzymatic activity and the selected thiol contents (**A**) and the controlling program of the microsolenoid devices (**B**). Abbreviations used in the figure: NO—normally open, NC—normally closed, P—solenoid micropump, V—solenoid microvalve, EI—enzyme-inhibitor mixing coil, PEDD—paired emitter-detector diode.

The developed analytical system consisted of three solenoid microvalves (V1, V2, V3), three solenoid micropumps (P1, P2, P3), a flow cell integrated with PEDD detector, a power source for the emitter diode, a controlling system (Arduino), and an ordinary multimeter. The system operation (presented in Figure 1) began with the alternate introduction of an enzyme and a phosphate buffer into the manifold using micropump P2 and microvalve V2. Thus, the normally closed (NC) position of the valve led the enzyme into the manifold, whereas the phosphate buffer was introduced via the normally open position (NO). The amount of time that the valve spent in the given positions was equal, which ensured the same volumes of reagents (160 µL) were injected into the system. At the same time, devices labeled as P1 and V1 enabled 320 µL of water (during the enzymatic activity determination) or the inhibitor (during the examination of selected thiols) to be provided to the system. The water introduced into the manifold by P1 and V1 also acted as a carrier stream (280 µL). Sample/inhibitor zones were precisely mixed and transported through the mixing coil EI (with a length of 30 cm and a volume of 230 µL) to the further part of the manifold. Devices P3 and V3 injected both the hydrogen peroxide and enzymatic substrate (pPD) into the system (each of 140 µL), where they were mixed with the sample/inhibitor. After reaching the detector’s flow cell via the reaction zone, the flow was stopped for a specific amount time, and a generated signal was recorded by an optoelectronic detection system. Subsequently, the flow was restored to start the cleaning procedure of the system in order to prepare it for the next measurement cycle. During this procedure, water and enzyme substrates were alternately injected into the system (the amount of each was 400 µL).

The photometric assay of the HRP activity, performed in the designed MCFA system, was based on the kinetic measurement of the analytical signal generated in the course of the bio-catalyzed oxidation of p-phenylenediamine (pPD) by hydrogen peroxide. The mentioned reaction leads to the creation of a purple product, the so-called Bandrowski’s base, which exhibits the absorption of about 530 nm [30], maximum. This maximum value is compatible with the maximum of the emission spectrum of the green LED applied in the developed PEDD-based detector as a light source. A red LED light detector was applied because LEDs are light-sensitive in narrow radiation ranges that are of higher energy than they emit [5]. The distance between these paired LEDs in the flow cell (path length) is 1.0 cm. Using a low-impedance voltmeter as an analytical signal recorder resulted in the increase of PEDD-detector sensitivity [6].

**Figure 2:**
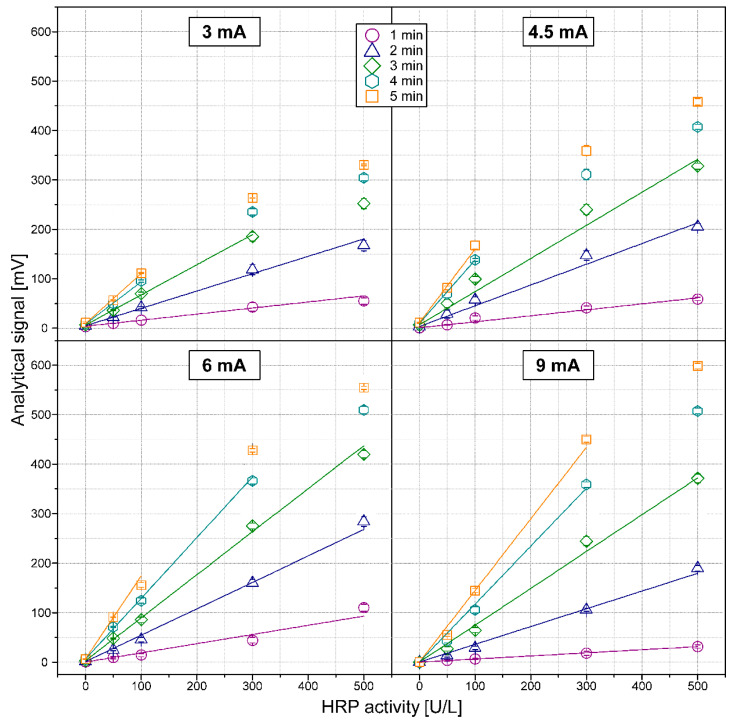
The detector responses for HRP activity in the range of 0–500 U/L depending on the currents supplying the emitter. The results are presented as analytical signals obtained for various incubation times. Error bars represent one standard deviation for *n* = 3.

The optimization the HRP assay procedures concerned both the performance of the detection system and the efficiency of an enzymatic reaction. First, the effects of the current supplying LED-emitter as well as incubation time between the enzymes and the substrates on the analytical signals (which can be defined as a subtraction between the baseline signal and the top of a negative peak) were examined. Measurements were conducted with 1.0 mmol/L pPD and 0.6 mmol/L H_2_O_2_. The obtained calibration graphs are shown in Figure 2. Increasing the current resulted in a sensitivity increase up to a certain value limit (in this case 6 mA) due to the characteristics of a LED–LED detector system [27]. The sensitivity of the 9-mA current in each case of incubation time was lower than for 6 mA. However, at the same time, it provided better precision (which generally affects the analytical performance of the method). The standard deviation for the analytical signal of a blank sample was 1.2 mV for 6 mA and only 0.2 mV for 9 mA. Moreover, the higher current affected the baseline (around 960 mV for 6 mA and 1180 mV for 9 mA), causing the wider dynamic range of the detector (Figure S2 in Supplementary Material). The LED power, which supplied the current of 9 mA and 4 min of incubation (as a compromise between the obtained sensitivity and the other analytical parameters, like time and reproducibility of analysis), was taken for further investigation.

**Figure 3:**
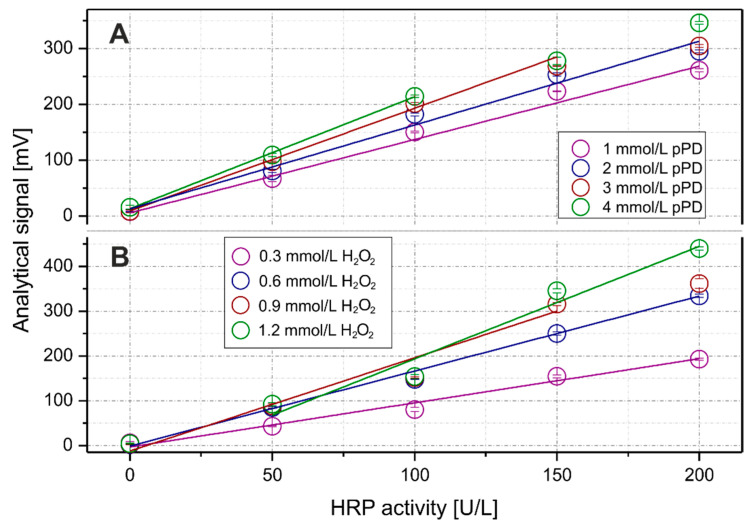
The optimization process of enzymatic reaction: the effects of pPD concentration (with 0.6 mmol/L H_2_O_2_, (**A**)) and hydrogen peroxide (with 2 mmol/L pPD; (**B**)) on obtaining analytical signals. Error bars represent one standard deviation for *n* = 3.

In addition, the effect of substrate concentrations was taken into account during the optimization process. As shown in Figure 3, the concentration increase of both the pPD and the hydrogen peroxide improved the sensitivity of the measurements. However, such effects had consequences that differed for each substrate. In case of the pPD, the increase of concentration decreased the upper limit of linearity (from 200 to 100 U/L), whereas the increase of the H_2_O_2_ concentration increased the lower limit of linearity (from 3.40 U/L for 0.6 mmol/L H_2_O_2_ up to 28.17 U/L for 1.2 mmol/L H_2_O_2_). As a compromise, 2.0 mmol/L of pPD and 0.6 mmol/L of H_2_O_2_ were considered optimal and were taken for further experiments.

**Figure 4:**
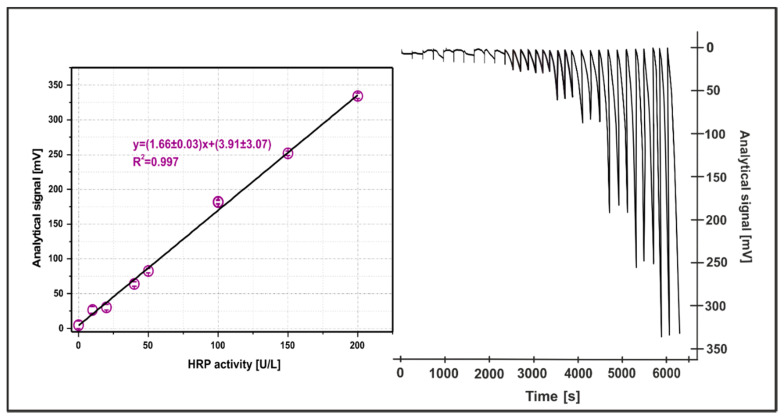
The recording of the calibration process for HRP activity in range of 0–200 U/L under the optimal conditions. The inset shows the corresponding calibration curve. Error bars represent one standard deviation for *n* = 3.

Figure 4 shows a typical recording and corresponding calibration graph obtained under optimal conditions. Negative peaks for HRP standards are related to a decrease of the light charging LED-detector, which was caused by an increase in the absorbance of the examined sample [27]. Moreover, the obtained recordings clearly showed that the baseline is stable over measurement times. The linear response of this system (y = (1.66 ± 0.03)x − (3.91 ± 3.07)) is in the range from 33.47 to 200 U/L for the HRP activity, with a coefficient of determination of 0.998. The presented method enabled the HRP activity to be determined with LOD and LOQ on the level of 10.54 U/L and 33.47 U/L, respectively. The LOD and LOQ have been calculated for 3 and 10 standard deviations signal values of 10 blank sample measurements, respectively. Furthermore, this method was characterized by a relatively high throughput—12 signal recordings per hour with acceptable precision (RSD below 6%).

**Figure 5:**
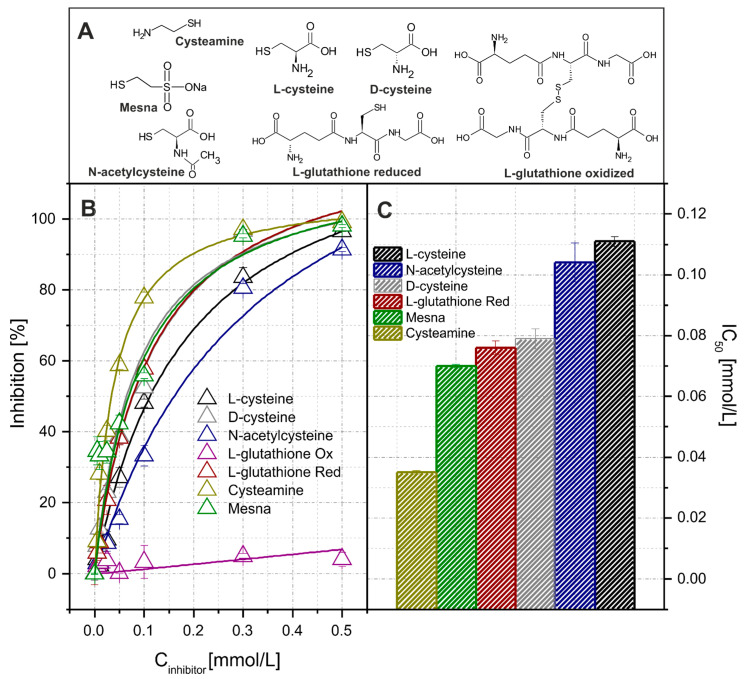
The structures of the selected thiols (**A**). The dependences between the reaction inhibition percentage and inhibitor concentration (**B**). The individual curves for each inhibitor are shown in Figure S4 in the Supplementary Materials. The column plot showing the IC_50_ (half maximal inhibitory concentration) of the particular thiols on HRP activity (**C**). The bar for the oxidized glutathione is not shown due to the lack of inhibitive activity. Error bars represent one standard deviation for *n* = 3.

The use of enzymatic assays for analytical purposes allows for the indirect quantitative determination of a wider group of analytes, including inhibitors. In this study, several simple thiols were chosen to demonstrate this ability (Figure 5). All further studies concerning thiol determination were conducted using 150 U/L HRP, 2.0 mmol/L pPD, and 0.6 mmol/L H_2_O_2_. As shown in Figure S3 (Supplementary Material), it was observed that the obtained signals were very similar for 0, 1, and 2 min of enzyme-inhibitor incubation in EI. This confirms that the inhibition of HRP by thiols is a rather fast process; therefore, further measurements were conducted without extra the stopping of the reacting segment. The inhibition percentage shown in Figure 5 was estimated as the ratio of difference between the signals obtained without and with the addition of the inhibitor. The curves shown in this figure were used as the calibration graphs for the selected thiols. Under given conditions, the effects from L-cysteine, D-cysteine, N-acetylcysteine, L-glutathione (reduced and oxidized), cysteamine, and mesna were investigated. Additionally, the IC_50_ values estimated for cysteamine, mesna, reduced L-glutathione, D-cysteine, N-acetylcysteine, and L-cysteine were 0.035, 0.070, 0.076, 0.079, 0.104, 0.111, respectively. It has been found that the analytical signal values correlate with the reducing reactivity of the tested thiols connected to some kind of substituents present in their structures. Nucleophilic, alkaline substituents increase the ability of the compounds to HRP inhibition. The substituent effects cause high cysteamine reactivity and low mesna reactivity, lower system sensitivity on N-acetylcysteine than D-cysteine, and the oxidized form of L-glutathione caused no inhibition of the biocatalyzed reaction, etc. Furthermore, it was found that an optical isomerism of cysteine has an influence on the inhibition process. In turn, the D-isomer of cysteine turned out to be a stronger HRP inhibitor than L-isomer. From the analytical point of view, it can be concluded that without any special optimization, the presented PEDD-MCFA system is sufficient for the determination of selected thiols (except oxidized L-glutathione and Mesna) with a LOD lower than 0.01 mmol/L. 

**Figure 6:**
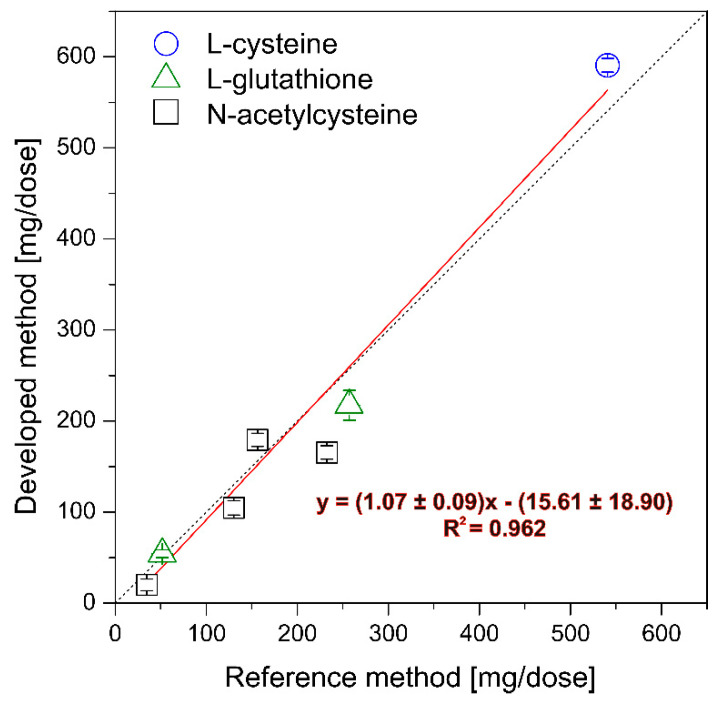
Correlation between the results obtained using the developed and reference method.

To demonstrate the utility of the developed PEDD-MCFA system for “real scenario” applications, the determinations of the selected thiols present in dietary supplements were performed. L-cysteine and L-glutathione are common antioxidant supplements, whereas N-acetylcysteine plays a double role: first, as a mucolytic drug and second, as a precursor of glutathione synthesis in the body. The results of the enzymatic analysis of such real samples were compatible with those obtained using pharmacopeally recommended iodometry (Figure 6). In some cases, a slight variation between the presented method and the reference method can be observed. The main reason for these deviations could be connected with the high inhomogeneity of some samples. In most cases, the results obtained by the iodometric method produced higher results than those of the MCFA/HRP system. This suggests that iodometry is less selective than the enzyme-inhibitive method due to the determination of the sum of reducing agents present in real samples and not only mercaptocompounds. The correlation between the reference method and the developed one for selected thiol content was the following: y = (1.07 ± 0.09)x − (15.61 ± 18.90), with the regression coefficient equal to 0.964. Furthermore, two-tail paired Student’s t-test (for 6 degrees of freedom and at the 95% confidence interval) also pointed out no statistically significant differences between the results of these methods. The calculated *t*-value (0.693) is appreciably lower than the tabulated value (2.447). Statistical results confirm the utility of developed bioanalytical MCFA system for the determination of thiol content such as L-cysteine, N-acetylcysteine, and L-glutathione (reduced) in some pharmaceutical products.

## 4. Conclusions

In the presented research, an efficient flow analysis system designed to detect horseradish peroxidase activity as well as its inhibitors was demonstrated. The flow analysis format of the bioassay has provided highly reproducible conditions for such kinetic measurements. This developed system is fully mechanized, economical, and easy to operate. Furthermore, the application of the optoelectronic detector allowed for the complete miniaturization of a system. The oxidoreductase detection principle in the flow analysis format proposed in this paper seems to be easily expanded to other kinds of biocatalytic analysis.

## Data Availability

Data is contained within the article or Supplementary Material.

## Supplementary Materials

The following are available online, Figure S1: 3D model of a flow-through photometric detector construction used in the course of presented studies for the determination of hrp activity and its inhibitors, Figure S2: The dependence between signal (uncorrected for baseline signal) and HRP activity for different emitter diode intensities obtained for 4 min of incubation time, Figure S3: The dependence between the inhibition and L-cysteine concentration for different incubation times of 150 U/L HRP with the inhibitor. The inset shows a magnification of the output plot in the concentration range between 0 and 0.1 mmol/L of L-cysteine (left). The corresponding graph shows the effect of the incubation time on the inhibition for different L-cysteine concentration (right). The error bars represent one standard deviation for *n* = 3, Figure S4: The dependence between the inhibition and inhibitor concentration, Table S1: Characteristics of studied dietary supplements and mucolytic drugs.

## Sample Availability

Samples of the compounds are not available from the authors.
