# Supplementary material for: Multicommutated Flow Analysis System for Determination of Horseradish Peroxidase and Its Inhibitors
Source: Molecules. 2021 Sep 16;26(18):5630. doi: 10.3390/molecules26185630 (PMC8465280; doi:10.3390/molecules26185630)
Supplement: Supplementary file 1 [file molecules-26-05630-s001.zip › molecules-1315045-supplementary.pdf]

molecules

MDPI

Multicommutated flow analysis system for determination of horseradish peroxidase and its inhibitors

Justyna Głowacka*, Kamil Strzelak and Robert Koncki

University of Warsaw, Faculty of Chemistry, Pasteura 1, 02-093, Warsaw, Poland

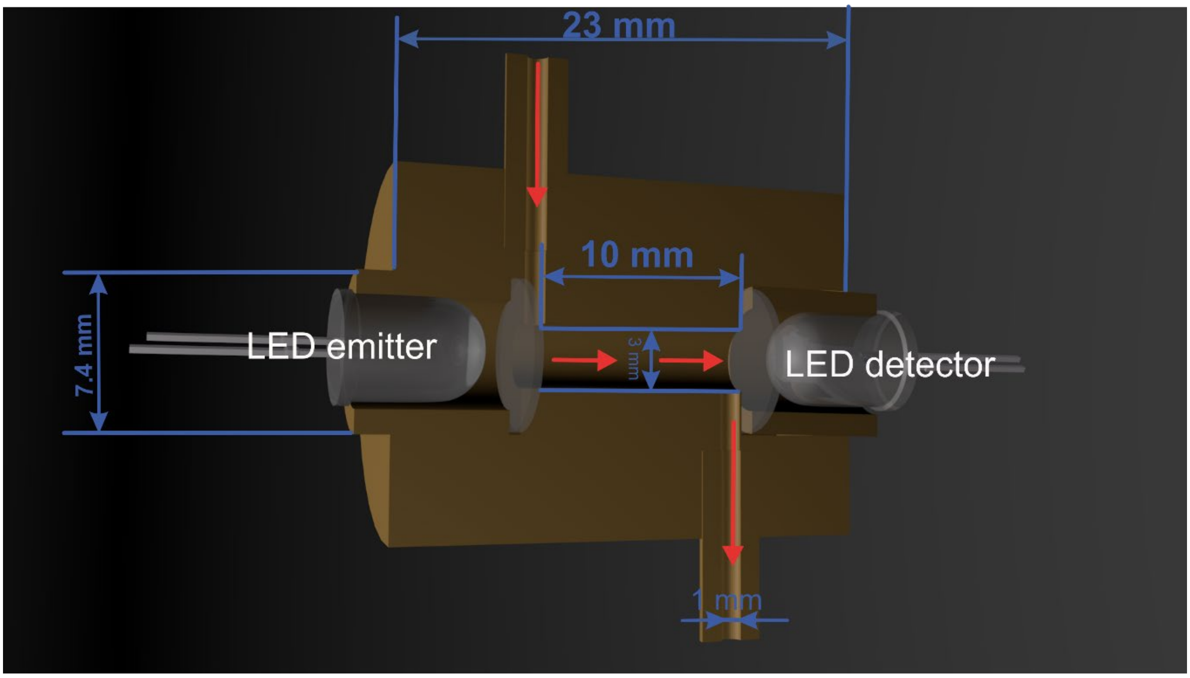

Figure S1. *3D model of a flow-through photometric detector construction used in the course of presented studies for the determination of hrp activity and its inhibitors.*

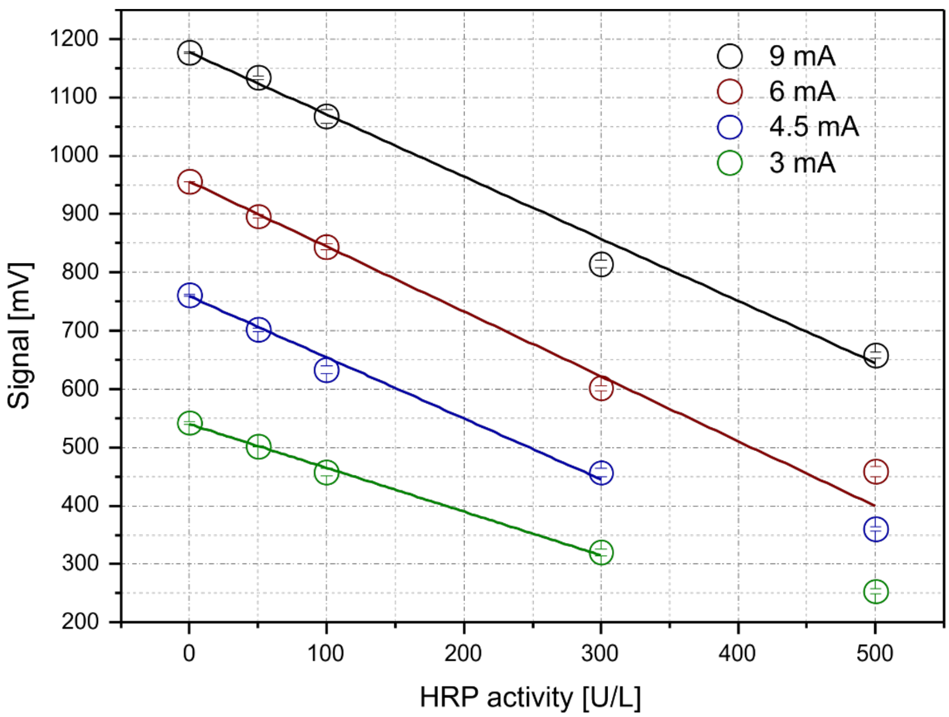

Figure S2. *The dependence between signal (uncorrected for baseline signal) and HRP activity for different emitter diode intensities obtained for 4 minutes of incubation time.*

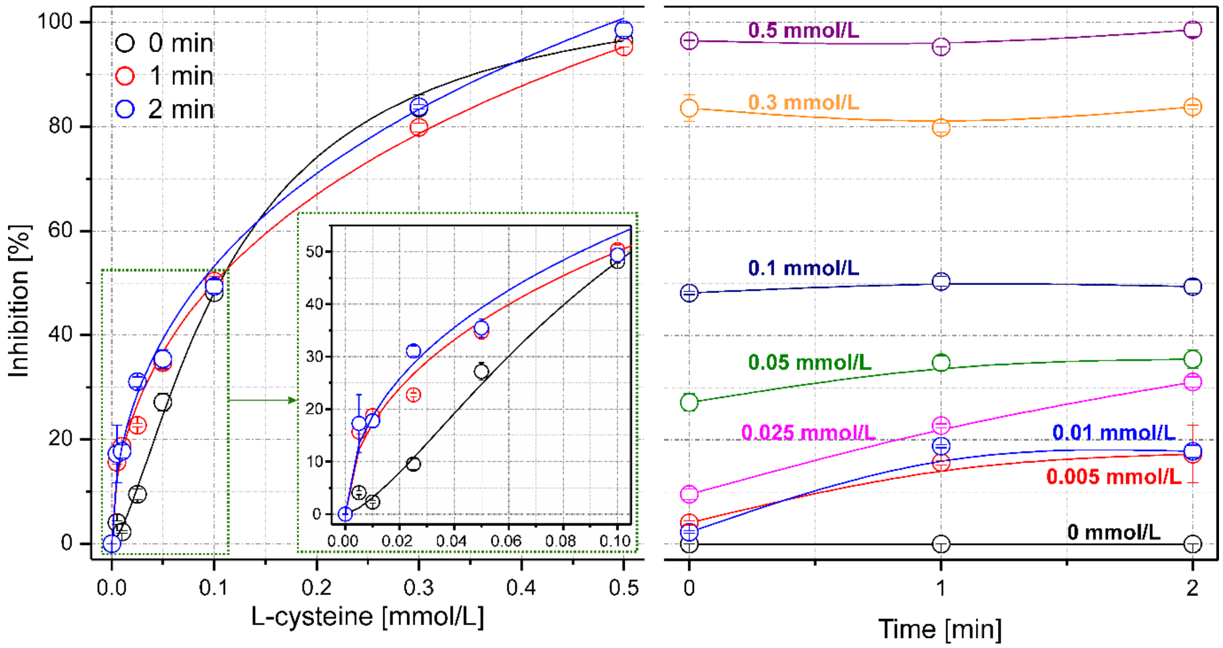

Figure S3. *The dependence between the inhibition and L-cysteine concentration for different incubation times of 150 U/L HRP with the inhibitor. The inset shows a magnification of the output plot in the concentration range between 0 and 0.1 mmol/L of L-cysteine (left). The corresponding graph shows the effect of the incubation time on the inhibition for different L-cysteine concentration (right). The error bars represent one standard deviation for n=3.*

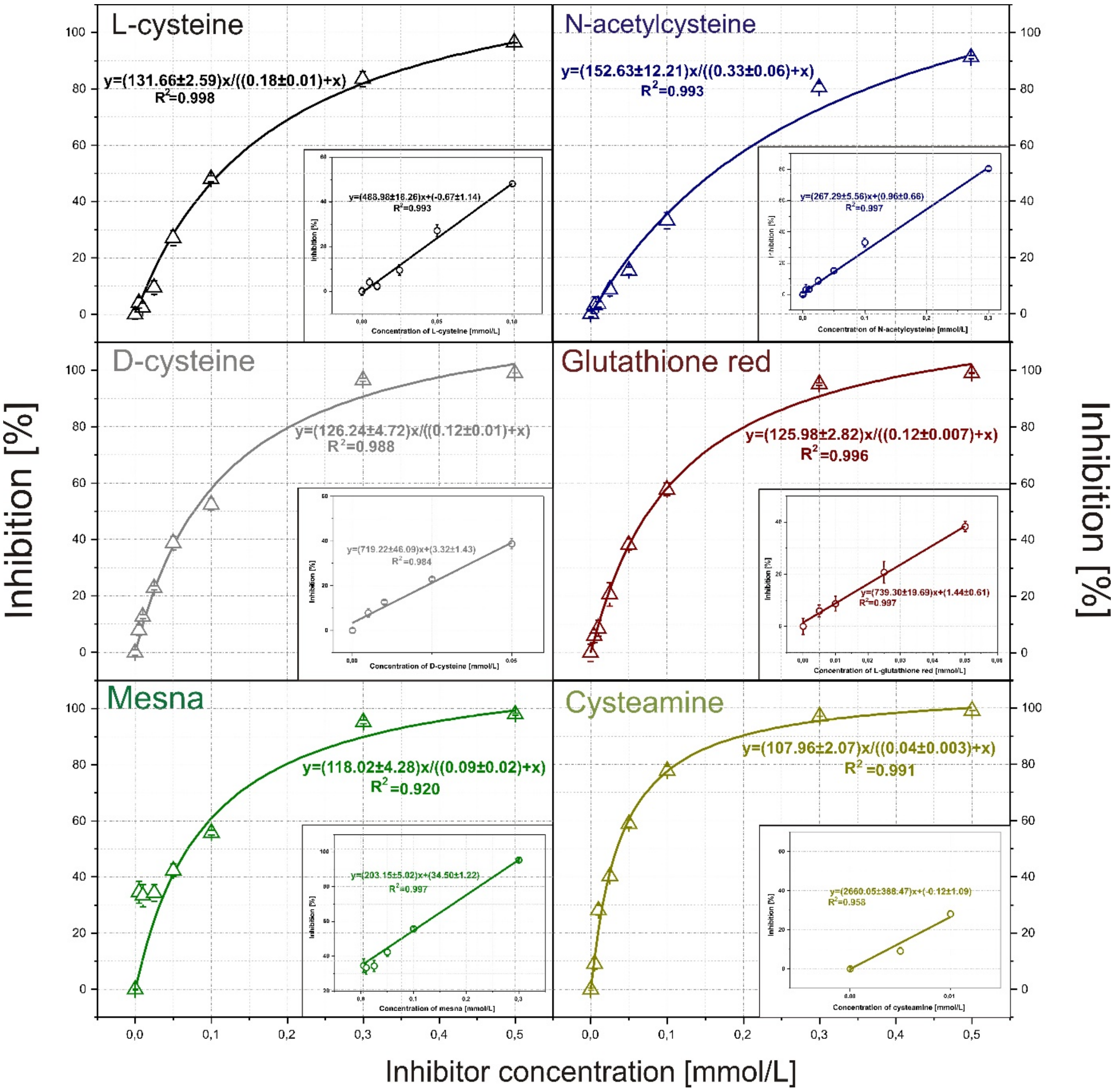

Figure S4. *The dependence between the inhibition and inhibitor concentration.*

Table S1. *Characteristics of studied dietary supplements and* mucolytic drugs.

Name	Producer	Thiol	Dosage form	Other main compounds	Declared content [mg/dose]
L-cysteine	Hanoju	L-cysteine	capsule with powder	hydroxypropyl methylcellulose	500
Glutathione reduced	Hanoju	L-glutathione (reduced)	capsule with powder	hydroxypropyl methylcellulose	250
Miositygon GT	Polski Lek		sachet with powder	Vitamins: B6, B9, B12, D, myo-inositol, manganese	50
ACC mini	Sandoz	N-acetylcysteine	sachet with powder	ascorbic and citric acids, mannitol, sodium bicarbonate	100
ACC classic	Sandoz		oral solution	sodium hydroxide, methylparaben, sodium benzoate	20
NAC	Kenayag		capsule with powder	hydroxypropyl methylcellulose	150
ACC	Sandoz		tablet	lactose, celulose, starch, citric acid, sodium cyclamate	200
